# Supplementary material for: Is ChatGPT 3.5 smarter than Otolaryngology trainees? A comparison study of board style exam questions
Source: PLoS One. 2024 Sep 26;19(9):e0306233. doi: 10.1371/journal.pone.0306233 (PMC11426521; doi:10.1371/journal.pone.0306233)
Supplement: S1 File — (DOCX) [file pone.0306233.s002.docx]

Question 1:

A 40 y.o male presents to the emergency room with concerns of right ear otalgia and otorrhea for the last week. He notes having greenish discolored drainage from his right ear. Which of the following microbes is the most likely cause of his infection?

1. Pseudomonas aeruginosa
2. Staphylococcus aureus
3. Streptococcus pneumoniae
4. Actinomyces Israeli
5. Enterococcus Faecalis

Correct Answer: A

Question 2:

The levator palpebrae superioris muscle is suspended by which of the following structures?

1. Medial canthal tendon
2. Lateral canthal tendon
3. Lockwood ligament
4. Whitnall ligament
5. Muller’s muscle

Correct Answer: D

Question 3:

A patient is seen in otology clinic with concerns of hearing loss. Audiogram is evident for a notch at 2000KHz. The patient’s father had some “stapes” surgery when he was a child which helped his hearing. During surgery, a blood vessel was noted running through the stapes footplate and the surgery was aborted, what is this structure derived from?

1. 1^st^ branchial arch
2. 2^nd^ branchial groove
3. 1^st^ branchial groove
4. 2^nd^ branchial arch
5. 1^st^ branchial pouch

Correct Answer: D – Middle meningeal A.

Question 4:

24-year-old male has a right sided vestibular neurofibroma, pigmented iris hamartomas, and hyperpigmented skin macules. The patient is diagnosed with a genetic condition. What chromosome is this defect located on?

1. 17
2. 23
3. 11
4. 15
5. 22

Correct Answer: 17 – NF1

Question 5

A 65-year-old female presents to your clinic after a Mohs resection of her basal cell carcinoma from her nasal tip and right nasal ala. The ablative surgeon had to resect more than initially anticipated leaving a large defect. You discuss with the patient a two-step reconstruction with a paramedian forehead flap. What is the ideal location and arterial pedicle of the flap?

1. Left sided flap based on the supratrochlear artery.
2. Right sided flap based on the supraorbital artery.
3. Left sided flap based on the angular artery.
4. Right sided flap based on the angular artery.
5. Right sided flap based on the supraorbital artery.

Correct answer: A – Paramedian flaps are based on the supratrochlear A

Question 6

A 13-year-old female with history of asthma which is well controlled with a leukotriene receptor agonist presents to the emergency room with a sore throat, runny nose, and nasal congestion. Which of the following virus is the most likely cause of her current condition?

1. Rhinovirus
2. Respiratory syncytial virus
3. Influenza virus
4. Parainfluenza virus
5. Herpes virus

Correct Answer: A – Rhinovirus for nasal symptoms.

Question 7

A 45-year-old female presents with chronic sialadenitis of her submandibular glands. Blood work confirms the presence of anti-SS-A and anti-SS-B antibodies. The patient should be counseled on an increased risk of which of the following malignancies?

1. Chronic lymphocytic leukemia
2. Chronic myeloid leukemia
3. Hodgkin’s lymphoma
4. Large B cell lymphoma
5. Mucosa-associated lymphoid tissue lymphoma

Correct answer – E – MALT

Question 8:

Which of the following is true of papillary thyroid carcinoma?

1. There is approximately 50% chance of having cervical metastatic disease at diagnosis in the general population.
2. This disease is associated with a TP53 mutation.
3. Children have a higher likelihood of having cervical metastasis compared to adults.
4. Patients will have higher levels of blood calcitonin levels.
5. It is associated with hyperparathyroidism.

Correct answer: C

Question 9:

A 2 year old male was brought to the emergency room after parents noted a significant cough episode while eating peanuts. Chest x-ray does not demonstrate a radiopaque object in the lungs. The patient is noted to have some expiratory stridor but is doing well otherwise. What is the next best step?

1. CT scan of the neck and chest
2. Flexible laryngoscopy
3. Operative bronchoscopy
4. Lateral chest x-rays
5. Heimlich maneuver

Correct answer: C

Question 10

What is the anterior boundary of the frontal recess?

1. Aggar nasi cell
2. Ethmoid bulla
3. Middle turbinate
4. Superior turbinate
5. Onodi cell

Correct answer: A

Question 11

A 60-year-old man was seen in the emergency room as a level 1 trauma consult. On a CT maxillofacial scan the patient is noted to have a left zygomaticomaxillary complex fracture along with a bony mass in the sinonasal tract. Which of the following sinuses are most frequently involved with osteomas?

1. Maxillary sinus
2. Anterior ethmoid sinus
3. Frontal sinus
4. Sphenoid sinus
5. Posterior ethmoid sinus

Correct answer: C

Question 12

A 25-year-old male is seen in the preoperative area and consent is being discussed with the patient as he will be having an awake laryngoscopy with excision of respiratory papillomatosis disease. You discuss the need to inject Bevacizumab (Avastin) after the laser ablation of the lesions. The patient asks what is the mechanism of action of Avastin?

1. PD-L1 inhibitor
2. VEGF inhibitor
3. RANKL inhibitor
4. mTOR inhibitor
5. TNF-Alpha inhibitor

Correct answer: B

Question 13

A 2-year-old male is seen in your clinic with concerns of chronic sinusitis. On anterior rhinoscopy there is evidence of bilateral nasal cavity polyposis. The mom states the patient had a history of delayed passage of meconium, but otherwise no relevant history was noted. Which of the following is the underlying mechanism of the likely diagnosis?

1. Mutation in a gene that regulates an electrolyte transporter protein.
2. Mutation in a gene that regulates mRNA silencing.
3. Mutation in a tyrosine kinase protein
4. Mutation in a NADPH oxidase protein
5. Mutation of a dynein arm protein.

Correct answer: A – CF – Chloride transporter protein.

Question 14

Which of the following is an indication for a parathyroidectomy procedure for a patient with asymptomatic primary hyperparathyroidism?

1. Bone mineral density reduced by more than 1 standard deviation by T-Score
2. The patient is younger then 50 years old.
3. Serum calcium level is 0.5 mg/dl greater than the upper limit of normal.
4. Creatine clearance is reduced by 20% for the age in absence of another cause.
5. 24 hours urine calcium level is 250 mg/dL.

Correct answer: B

Question 15

A 57-year-old male is scheduled for a bicoronal procedure to resect an esthesinoneuroblastoma from the anterior cranial vault. The anterior skull base reconstruction was to be performed with a temporalis muscle flap, however, the resident accidently cut both superficial temporal arteries. What is the next best step?

1. Continue with the planned temporalis muscle flap.
2. Radial forearm free flap
3. Latissimus dorsi free flap
4. Paramedian forehead flap
5. Auricular cartilage graft.

Correct answer – A – Temporalis flap is based on the deep temporal A not superficial.

Question 16

Which of the following is true of primary hyperparathyroidism?

1. Is caused by one or more parathyroid adenoma in only 20% of cases.
2. Patients will have an increase in urine phosphate levels
3. Patients will have an increase in urine sodium levels
4. Patients will have normal PTH (parathyroid hormone) levels
5. Is commonly caused by a parathyroid carcinoma.

Correct answer: B

Question 17

A 24-year-old female presents for follow up after having sinus surgery last week. She notes having clear rhinorrhea that worsens with leaning over. This fluid was sent for analysis and is confirmed to be cerebrospinal fluid (CSF). Which of the following location is the likely cause of this iatrogenic CSF leak?

1. Fovea ethmoidalis
2. Sphenoid sinus
3. Posterior table of the frontal sinus
4. Lamina papyracea
5. Maxillary sinus

Correct answer: A

Question 18

Which of the following findings would require an intraoperative neck dissection during a parotidectomy?

1. A papillary cystadenoma lymphomatosum tumor on frozen section.
2. A basal cell carcinoma
3. An adenoid cystic carcinoma involving the facial nerve.
4. A high grade mucoepidermoid carcinoma
5. A pleomorphic adenoma

Correct answer: D

Question 19

A 30 year old male with long standing history of sinonasal disease presents to clinic with a nasal congestion, thick purulent nasal discharge, headaches, facial pain, and fevers. Culture of the drainage will likely grow which of the following bacteria?

1. Helicobacter pylori
2. Moraxella catarrhalis
3. Bacteroides fragilis
4. Streptococcus pneumoniae
5. Staphylococcus epidermidis

Correct answer: D

Question 20

A 70 year old male with history of kidney transplant presents to the emergency room with headaches and purulent nasal drainage. CT scan is evident for right maxillary sinusitis. Nasal endoscopy is evident for black eschar of the middle turbinate. Biopsy of this area is consistent with septate hyphae with 45 degree branching pattern. Which of the following is the correct treatment?

1. IV Vancomycin
2. IV Voriconazole
3. IV metronidazole
4. IV Amphotericin
5. IV ampicillin-sulbactam

Correct answer – B – Voriconazole tx aspergillus invasive fungal sinusitis (45 degree) vs amphotericin which treats mucormycosis (nonseptate and branches at 90 degrees)

Question 21

A 50 year old male recently had a tracheostomy tube placed in the operating room after being intubated for 14 days due to a severe COVID infection. The patient is now having significant bleeding from the tracheostomy site and a trachea-innominate artery fistula is suspected. What is the first step in management?

1. Hyperinflate the tracheostomy balloon cuff.
2. Emergent blood transfusion.
3. Place your finger into the stoma to hold pressure.
4. Emergent CT angiography.
5. Remove the tracheostomy tube.

Correct answer – A – prevent blood going into the lungs.

Question 22

What subtype of basal cell carcinoma is more aggressive mandating a larger resection?

1. Nodular
2. Sclerosing
3. Superficial
4. Deep
5. Pigmented

Correct answer: B

Question 23

A 45 year old female is seen in laryngology clinic with recent diagnosis of sarcoidosis by her pulmonologist. Flexible laryngoscopy may demonstrate a lesion in which of the following structures?

1. Nasopharynx
2. True vocal fold
3. Subglottic airway
4. Epiglottis
5. Piriform recess

Correct answer: D

Question 24

A 45-year-old male from presents to your clinic for evaluation of a nasal mass. He recently immigrated from Israel to the United States. Biopsy of the nasal mass demonstrates foamy histiocytes with a “moth-eaten” appearance of the cytoplasm. Additionally, there are large plasma cells with birefringent inclusions. What is the diagnosis?

1. Squamous cell carcinoma
2. Basal cell carcinoma
3. Hodgkin’s lymphoma
4. Rhinoscleroma
5. Rhinosporidiosis

Correct answer: D

Question 25

A 74-year-old female is evaluated in a neurology clinic for fatigue and double vision. She notes her double vision starts in the evenings with associated eyelid drooping. Extensive workup shows an antibody against the acetylcholine receptors. Chest x-ray demonstrates a widened mediastinum suggestive of a thymoma. What pharyngeal pouch does the thymus derive from?

A) 1^st^

B) 2^nd^

C) 3^rd^

D) 4^th^

E) 5^th^

Correct answer: C

Question 26:

A 12-year-old female is seen in clinic with concerns of sleep disturbances. Patient describes having repetitive leg twitching causing poor sleep and fatigue during the day. A sleep study demonstrates twitching of the anterior tibialis muscle. What is the correct diagnosis?

1. Primary idiopathic hypersomnolence
2. Central obstructive sleep apnea
3. Restless leg syndrome
4. Periodic limb movement disorder
5. Insufficient sleep syndrome

Correct answer: D – RLS is subjective tingling of the lower legs relieved by movement, whereas PLMD is objective movement of ATM seen on sleep study.

Question 27

A 4 year old male is seen in an otolaryngology clinic with concerns for obstructive nasal breathing. Operative biopsy of the lesion confirms the diagnosis. Pathology is concerning a poorly differentiated lesion with Z bands. What is the diagnosis?

1. Juvenile Nasopharyngeal Angiofibroma
2. Chordoma
3. Lymphoma
4. Rhabdomyosarcoma
5. Squamous cell carcinoma

Correct answer: D

Question 28

A 28-year-old female presents to your clinic with concerns of voice changes. Upon hearing her voice, you notice occasional breaks in her voice during speech. She states her voice gets better when going out with friends. Flexible laryngoscopy confirms the diagnosis. Which of the following medications can help with her voice?

1. Propranolol
2. Botulism toxin
3. Methocarbamol
4. Dexamethasone
5. Carvedilol

Answer: B

Question 29

A 45 year old male was seen in the emergency room after being assaulted in the bar. A CT scan of the temporal bones is evident for a otic capsule involving fracture. The patient has a facial nerve palsy of house Brackman VI/VI. Which of the following area along the course of the facial nerve is likely involved in the fracture?

1. The cerebellopontine angle
2. The perigeniculate region
3. As it exits the stylomastoid foreman
4. The vertical segement of the facial nerve
5. Within the internal auditory canal.

Answer: B

Question 30

A nurse is having difficulty passing a nasogastric tube into a premature infant in the NICU. A bedside flexible laryngoscopy confirms the diagnosis. Which of the following are associated with the above diagnosis?

1. Thymic aplasia
2. Cleft palate
3. Cleft lip
4. Coloboma
5. Hyperkalemia

Answer: Coloboma (CHARGE syndrome)
