# Supplementary material for: Is ChatGPT 3.5 smarter than Otolaryngology trainees? A comparison study of board style exam questions
Source: PLoS One. 2024 Sep 26;19(9):e0306233. doi: 10.1371/journal.pone.0306233 (PMC11426521; doi:10.1371/journal.pone.0306233)
Supplement: Attachment — Submitted filename: Response To Reviewers.docx [file pone.0306233.s003.docx]

Dear Reviewers,

Thank you for your evaluation of our study. We appreciate your time to review our work and provide your feedback. We hope we have addressed all your concerns in our revised manuscript. We will also provide our responses to your questions outlined below:

Reviewer #1: The article compared the performance of ChatGPT with that of human. Simple and straightforward study. Just need to cite some more recent literature in the discussion like <https://ijvlms.sums.ac.ir/article_49682.html>. The version of the chatGPT can also be mentioned in the title of the article so that readers understand at a glance which chatgpt you are referring to.

-Thank you, we updated our title and reviewed the provided study and incorporated into our discussion.

Reviewer #2: Thank you for this interesting paper. While the results no doubt add to our understanding of AI in medicine, there are a few comments to be made:

1) You do not provide any information about the questions asked of the two groups. How were they constructed? Are the questions at different levels or graded, and how do we know that? The quality of the questions and answers no doubt have implication on the results and should be shared with the reader. This is particularly given your conclusion that ChatGPT struggles with harder questions, but we don't have the data to know that's where it struggled or that's where higher learners didn't struggle. Also, do we have a comparison to know that higher learners do this well on these tests in general? Is a score of 85% expected from PGY5s?

-Thank you, we drafted these questions from a previously published bank of board prep questions. We generated the 30 questions with highlighting easy, moderate, and difficult questions encompassing all subspecialities of otolaryngology. We did alter minute details from each question without changing its underlying stem or degree of difficulty of the question to prevent infringement of this question bank. Unfortunately, we do not have the data demonstrating which question is easy, moderate, of difficult to run a statistical analysis on which questions were more frequently missed. We did compare the historical pass rate of senior residents on the board exam and our questions do appear to demonstrate an average that would be appropriate for a level of training.

2) The article suggests you asked ChatGPT to provide an answer and then explain their reasoning. It does not appear this was done for human participants. Why is that? Your article suggests there were times that the answers and the explanations did not match. Could this also not be the case for humans? Again, also knowing the questions could provide insight here. Were there certain questions that were more likely to trip up humans or ChatGPT?

-Thank you, we did not ask ChatGPT to give us an explanation for its answers. This was an incidental finding that we reported on as it was an interesting find. You can argue that humans do the same when guessing which we think is interesting to see an AI appear as if it’s guessing. We did not perform this analysis to see if certain questions were more likely to cause errors as this was not the intended goal of our study.

3) What is the denominator of participants? How many people were invited to participate? Is there potential bias introduced based on who participates?

-Thank you, we provided the denominator of participants and the total number of individuals invited. We attempted to avoid bias and this was addressed in our study.

4) Please include potential issues with this study in your discussion.

-Thank you, we did highlight the lack of participants being a limitation in our study.

5) Your discussion includes a paragraph about the ethical implications of AI. It does seems to fit with the paper itself (ie the study doesn't focus on AI ethics) nor are there any references provided. You may want to remove this, and from the conclusion.

-Thank you, this was reflected.

Reviewer #3: This is a well written but not rigorously conducted study on the test taking abilities of chatgpt3.5. The strength of the paper Include its simply and coherently written introduction and methods. The weakness of this paper is in the rigor of the experiment design and the construction of the test for chatgpt. Firstly, it is unclear whether the 30 questions chosen are valid representations of otolaryngology board examination questions. There is no explanation of how they were chose, how they were vetted and against what criteria, and what mix of topics/difficulty they test. Many articles have been written on similar topics that can be referenced for their methodology. (Ideally, the questions used would be real board questions, but in the absence of that, there are still ways to objectively vet and classify questions. For example, see <https://pubs.rsna.org/doi/10.1148/radiol.230582>).

- Thank you, we drafted these questions from a previously published bank of board prep questions. We generated the 30 questions with highlighting easy, moderate, and difficult questions encompassing all subspecialities of otolaryngology. We did alter minute details from each question without changing its underlying stem or degree of difficulty of the question to prevent infringement of this question bank.

Secondly, the discussion does not root the paper within the existing literature, with zero citations or references to other similar work. The discussion actually begins to present new information (anecdotal observations of how chatgpt is reasoning through questions or not) that was not shown in results

-Thank you, this was addressed with addition of different studies in the discussion and how they related to our study. We also changed the flow of our discussion to summarize our findings.

Lastly, the authors should try alternative prompts to see if chatgpt may Perform better if, for example, it was told it is a practicing otolaryngologist rather than a medical professional, etc.

-Thank you, we tried to keep the repertoire of knowledge broad to the whole of medicine by keeping it a medical professional rather than an otolaryngologist to prevent bias in its data. This could be something we could look into future studies to see if prompting ChatGPT differently results in different answers to the same questions.
